# *Chlorella* sp. and *Nannochloropsis* sp. Inclusion in Plant-Based Diets Modulate the Intestine and Liver Antioxidant Mechanisms of European Sea Bass Juveniles

**DOI:** 10.3389/fvets.2020.607575

**Published:** 2020-12-17

**Authors:** Carolina Castro, Filipe Coutinho, Paula Iglesias, Aires Oliva-Teles, Ana Couto

**Affiliations:** ^1^Centro Interdisciplinar de Investigação Marinha e Ambiental (CIIMAR/CIMAR), Terminal de Cruzeiros do Porto de Leixões, Matosinhos, Portugal; ^2^Buggypower, Parque Industrial Base 2000, Lorquí-Murcia, Spain; ^3^Departamento de Biologia, Faculdade de Ciências da Universidade do Porto, Porto, Portugal

**Keywords:** oxidative stress, fish health and welfare, fish meal replacement, antioxidants, microalgae, alternative ingredients

## Abstract

This study aimed to evaluate the effects of including microalgae *Chlorella* sp. or *Nannochloropsis* sp. in plant-based diets on antioxidant mechanisms of European sea bass (*Dicentrarchus labrax*) juveniles. For this purpose, three isoproteic (50%) and isolipidic (19%) diets were formulated: a practical diet, containing 15% fish meal (FM) and plant ingredients as the protein source and a mixture of fish oil and vegetable oils (40: 60) as lipid source (control diet); and two diets identical to the control but with the FM replaced by *Nannochloropsis* sp. or *Chlorella* sp. (diets Nanno and Chlo, respectively). The diets were offered to quadruplicate groups of 25 fish (initial body weight: 24 ± 1 g) for 11 weeks and then enzymatic and non-enzymatic antioxidant mechanisms and lipid oxidative biomarkers were assessed in the liver and intestine of these fish. Results showed that the antioxidant response was tissue-dependent, with the liver exhibiting lower glutathione peroxidase and glucose-6-phosphate dehydrogenase (only in Chlo group) activities, and intestine lower superoxide dismutase activity with the diets including microalgae compared to control diet. An increase of oxidized glutathione content was also observed in the intestine of fish fed the microalgae diets. Catalase and glutathione reductase activities, oxidative stress index, and total and reduced glutathione, were unaffected by dietary treatments in both tissues. Overall, the lipid peroxidation status was not compromised by the replacement of FM by microalgae.

## Introduction

Finding sustainable ingredients alternative to the finite world fisheries products remains a major issue for the sustained growth of aquaculture (1). Plant feedstuffs (PF, such as soybean meal, corn gluten meal, rapeseed meal), due to their abundance, relatively constant availability, and affordable prices, have been used as the main alternative protein and lipid sources in aquafeeds, including for carnivorous species (2). Although high or total replacement of fish meal (FM) by PF without affecting growth performance has been successfully achieved in carnivorous species, the presence of antinutritional factors (protease inhibitors, lectins, saponins, phytic acid, non-starch polysaccharides, among others) in most PF has been related to adverse effects on fish nutrient digestion and utilization, liver and intestine histomorphology, immune, and inflammatory responses (3–6). Overall, this may affect target functions in the body, including increased fish susceptibility to oxidative stress.

Oxidative stress occurs when the balance between the generation of reactive oxygen species (ROS)–derived directly as by-products of aerobic metabolism, fatty acids peroxidation, or promoted by external factors (temperature, handling, nutrition)–exceeds that of ROS removal, thus inducing tissue oxidative damage (7). Fish, being rich in *n*−3 long-chain polyunsaturated fatty acids (LC-PUFA), are particularly vulnerable to ROS attack, which can lead to lipid peroxidation (LPO) (7–9). To prevent oxidative injury, animals developed a complex antioxidant protection system that involves antioxidant enzymes (superoxide dismutase, SOD; catalase, CAT; glutathione peroxidase, GPX; glutathione reductase, GR) and non-enzymatic antioxidants (Coenzyme Q10, glutathione, ascorbic acid, uric acid, lipoic acid, bilirubin, etc.) (7).

Given the potential negative effects of PF-based diets, there is a continuous search for novel alternative dietary protein and lipid sources to include in aquafeeds and of ingredients that mitigate the susceptibility for increased oxidative stress. In this context, microalgae have recently gained increasing attention. Depending on the species and culture/harvesting conditions, dry microalgae biomass are characterized by medium to high protein (30 to 70% dry matter-DM) and lipid (10 to 20% DM) levels, as well as proper amino acid and fatty acid composition (10). Several microalgae species (such as *Tetraselmis* sp., *Nanofrustulum* sp., and *Tisochrysis* sp.) proved adequate to partially replace FM in diets for carnivorous fish species without negative effects on growth performance (11–13). Besides their value as protein and lipid sources, microalgae are also being considered for their health-promoting potential, as they are a rich source of bioactive compounds, such as pigments, vitamins, minerals, and antioxidants (14). These bioactive compounds are reported to have a high spectrum of biological functions, including anti-inflammatory, immunomodulatory, antioxidant, and antibacterial activities (15–17).

Although microalgae are considered a natural source of antioxidants, evidence of their effects on the fish antioxidant system is still scarce and controversial. For instance, in turbot (*Scophthalmus maximus*), the inclusion of up to 5% of *Nannochloropsis* sp. in diets, replacing 7.8% of FM, enhanced the fish antioxidant capacity, by decreasing serum LPO and increasing serum and liver SOD and GPX activities (18). In Atlantic salmon (*Salmo salar*), replacement of 20% of dietary FM with defatted *Desmodesmus* sp. or *Nannochloropsis oceania* biomass did not affect serum SOD and CAT activities (19, 20). Furthermore, in olive flounder (*Paralichthys olivaceus)* dietary inclusion of 10–15% defatted *Chlorella vulgaris* did not affect plasmatic GPX and SOD activities but increased CAT activity (21).

In the aforementioned studies, the antioxidant potential of microalgae was mostly assessed with diets including high levels of FM and fish oil(FO) and focused on serum or liver antioxidant (18–21). Up to now, little attention was given to the antioxidant potential of dietary microalgae when incorporated into PF-based diets, nor their effects at the intestine level, which is the first organ exposed to the diets. The intestine is highly sensitive to factors inducing oxidative stress, and imbalances in the redox state have important implications in its functionality and health (22).

Thus, the present study aimed to evaluate the effects on the intestine and liver oxidative system of including the microalgae *Chlorella* sp. or *Nannochloropsis* sp. in practical diets for the European sea bass (*Dicentrarchus labrax*), an economically important carnivorous fish species of Mediterranean aquaculture.

*Chlorella* and *Nannochloropsis* were the microalgae chosen for this study as they are amongst the most used in aquaculture feeds as they have high protein content (23). These microalgae are also good sources of *n*−3 LC-PUFAs, which are nutritionally required by marine fish and are important for human health (14, 23). Thus, these microalgae could be considered not only a promising alternative protein source to conventional protein sources (such as plant proteins and fish meal) as well as an alternative to traditional lipid sources as FO.

## Materials and Methods

The study was approved by the ORBEA Animal Welfare Committee of CIIMAR and the Portuguese National Authority for the Animal Health (DGAV). It was directed by certified scientists (Functions A, B, C, & D defined in article 23 of European Union Directive 2010/63), and all procedures were conducted according to the Federation of Laboratory Animal Science Association (FELASA) recommendations and the EU Directive (2010/63/EU) on the protection of animals for scientific purposes.

### Diets

Three experimental diets were formulated to contain 50% protein and 19% lipids. Also, diets were formulated to fulfill the species aminoacids requirements and to be isoaminoacidic. A control diet was formulated to include circa 20% protein from FM and 80% from PF, and a fish oil/vegetable oil mix (40: 60) as lipid source. This diet was formulated to be a practical diet as current commercial diets for European sea bass already includes only 15% FM. To test the feasibility of total replacement (15%) of dietary FM by microalgae, two other diets were formulated similar to the control but without FM, which was replaced by *Chlorella* sp. (diet Chlo) or *Nannochloropsis* sp. (diet Nanno). Dietary protein content was adjusted by manipulating wheat meal and faba beans levels.

The lyophilized microalgae were provided by Buggypower, Lda. (Porto Santo, Portugal) and were produced in a marine environment and in a completely autotrophic closed production system called photobioreactors. Biomass was collected, centrifuged, freeze-dried, vacuum stored in plastic bags, and refrigerated until used. Microalgae composition is detailed in Table 1. Diets were manufactured by SPAROS (Olhão, Portugal) using a pilot-scale twin-screw extruder (CLEXTRAL BC45, France) to a pellet size of 2 mm, and oil was added after the extrusion process by means of vacuum coating (Dinnissen Pegasus vacuum mixer, PG-10VCLAB, Netherlands). All batches of extruded feeds were dried in a convection oven (OP 750-UF, LTE Scientifics, United Kingdom) and stored at 4°C until use. Ingredients and proximate composition of the diets are presented in Table 2.

**Table 1:** Proximate composition (% dry matter) of microalgae.

	***Nannochloropsis* sp**.	***Chlorella* sp**.
Dry matter (%)	96.4	91.9
Protein	47.5	48.2
Lipids	9.2	12.3
Carbohydrates	28.7	15.0
Ash	11.0	16.4
**Fatty acid composition (% FA)**		
Total saturated fatty acids (SFA)	42.3	19.7
Total monounsaturated fatty acids (MUFA)	8.8	10.0
Total polyunsaturated fatty acids (PUFA; AA, EPA, DHA^*^)	48.3	70.0
Other fatty acids	0.6	0.3
Σɷ3	35.2	35.6
Σɷ6	8.6	19.4
Σɷ3/ɷ6	4.1	1.8

* *AA, Arachidonic Acid; EPA, Eicosapentaenoic Acid; DHA, Docosahexaenoic Acid*.

**Table 2:** Ingredient composition and proximate analysis of the experimental diets.

	**Control**	**Nanno15**	**Chlo15**
**Ingredients (%)**			
Fishmeal^a^	15.0	–	–
Nannochloropsis^b^	–	15.0	-
Chlorella^c^	–	–	15.0
Soy protein concentrate^d^	14.8	14.8	14.8
Pea protein concentrate^e^	2.0	2.0	2.0
Wheat gluten^f^	6.0	12.3	12.5
Corn gluten^g^	14.7	14.7	14.7
Soybean meal 48^h^	7.5	7.5	7.5
Rapeseed meal^i^	7.5	7.5	7.5
Wheat meal^j^	3.3	2.9	3.1
Faba beans (low tannins)^k^	10.0	4.0	4.0
Fish oil^l^	5.8	5.2	4.8
Rapeseed oil^m^	8.7	8.7	8.7
Vitamin and mineral premix^n^	1.0	1.0	1.0
Betaine HCl^o^	0.1	0.1	0.1
Soy lecithin (powder)^p^	1.0	1.0	1.0
Binder^q^	0.4	0.4	0.4
Antioxidant powder^r^	0.2	0.2	0.2
Sodium propionate^s^	0.1	0.1	0.1
Monoammonium phosphate (MAP)^t^	1.0	1.1	1.1
L-Lysine^u^	0.4	0.8	0.8
L-Threonine^v^	0.1	0.1	0.1
DL-Methionine^w^	0.4	0.6	0.6
**Proximate analysis (% dry weight)**			
Dry matter (DM)	92.2	87.9	87.1
Crude protein	49.6	49.1	51.8
Crude fat	20.3	19.9	18.9
Ash	7.0	6.0	8.3
Gross energy (KJ g-1 DM)	22.5	23.0	22.2
**Fatty acid composition (% of total fatty acids)**			
Σ SFA	17.0	17.6	16.7
Σ MUFA	48.2	47.2	47.3
Σ *n*-6	17.42	18.23	19.45
Σ *n*-3	14.9	14.4	13.9
**Ratios**			
*n*-6/*n*-3	1.17	1.27	1.40
SAT/PUFA	0.52	0.53	0.50

a *NORVIK 70: 70.6% crude protein (CP), 5.8% crude fat (CF), Sopropêche, France*.

b,c *Freeze-dried biomass: proximal composition described in Table 1, Buggy Power, Madeira Island, Portugal*.

d *Soycomil: 63% CP, 0.8% CF, ADM, The Netherlands*.

e *Lysamine: 86% CP, 1% CF, ROQUETTE Frères, France*.

f *VITAL: 83.7% CP, 1.6% CF, ROQUETTE Frères, France*.

g *61% CP, 6% CF, COPAM, Portugal*.

h *Solvent extracted dehulled soybean meal: 47% CP, 2.6% CF, CARGILL, Spain*.

i *Defatted rapeseed meal: 37.7% CP, 2.3% CF, Premix Lda, Portugal*.

j *10.2% CP; 1.2% CF, Casa Lanchinha, Portugal*.

k *Faba beans: 28.5% CP; 1.2% CF, Ribeiro & Sousa Cereais, Portugal*.

l *SAVINOR UTS, Portugal*.

m *Henry Lamotte Oils GmbH, Germany*.

n *PREMIX Lda, Portugal: Vitamins (IU or mg/kg diet): DL-alpha tocopherol acetate, 100 mg; sodium menadione bisulphate, 25 mg; retinyl acetate, 20,000 IU; DL-cholecalciferol, 2,000 IU; thiamin, 30 mg; riboflavin, 30 mg; pyridoxine, 20 mg; cyanocobalamin, 0.1 mg; nicotinic acid, 200 mg; folic acid, 15 mg; ascorbic acid, 500 mg; inositol, 500 mg; biotin, 3 mg; calcium panthotenate, 100 mg; choline chloride, 1,000 mg, betaine, 500 mg. Minerals (g or mg/kg diet): copper Sulfate, 9 mg; ferric Sulfate, 6 mg; potassium iodide, 0.5 mg; manganese oxide, 9.6 mg; sodium selenite, 0.01 mg; zinc Sulfate,7.5 mg; sodium chloride, 400 mg; excipient wheat middling's*.

o *ORFFA, The Netherlands*.

p *Lecico P700IPM, LECICO GmbH, Germany*.

q *Kieselguhr, LIGRANA GmbH, Germany*.

r *Paramega PX, Kemin Europe NV, Belgium*.

s *Disproquímica, Portugal*.

t *Windmill AQUAPHOS: 26% P, Aliphos, The Netherlands*.

u *L-Lysine HCl 99%: Ajinomoto Eurolysine SAS, France*.

v *ThreAMINO 98.5%, Evonik Nutrition & Care GmbH, Germany*.

w *DL-METHIONINE FOR AQUACULTURE 99%, EVONIK Nutrition & Care GmbH, Germany*.

*SFA, saturated fatty acids; MUFA, monounsaturated fatty acids; PUFA, polyunsaturated fatty acids; n−3 LC-PUFA, n−3 long chain polyunsaturated fatty acids*.

### Feeding Trial

The trial was carried out at the experimental facilities of the Marine Zoology Station, Porto University. European sea bass (*Dicentrarchus labrax*) juveniles with average initial body weight (IBW) of 24 ± 1 g were obtained from a commercial hatchery (Acuinuga, Spain) and kept in quarantine for 3 weeks. During this period fish were fed a commercial diet (AquaGold, Aquasoja; Sorgal, S.A., Portugal).

Thereafter, homogeneous groups of 25 fish were randomly distributed to 12 rectangular fiberglass tanks of 100 L water volume in a thermo-regulated recirculating water system, supplied with a continuous flow of filtered seawater and aeration provided by diffusion through air stones. Each experimental diet was randomly assigned to quadruplicate groups and the fish were fed by hand, twice a day, 6 days a week, until apparent visual satiation. Utmost care was taken to ensure that all feed supplied was consumed. The trial lasted 11 weeks and during this period water temperature averaged 23.0 ± 0.5°C, salinity was maintained at 35,000 ± 1,000 mg/L, and dissolved oxygen was kept near saturation (7.0 mg/L). The photoperiod regime adopted was 12:12 h light: dark, provided by artificial illumination.

### Sampling

At the end of the trial, three fish from each tank were randomly collected 3 h after the last meal as described in Couto et al. (24), and euthanized by severing the spinal cord. Sampled fish were dissected on chilled trays, the digestive tract excised and freed from adjacent adipose and connective tissues, and the liver and intestine removed and rapidly frozen in liquid nitrogen and then stored at −80°C until analysis.

### Chemical Analysis

Dry matter, crude protein, crude lipids, gross energy, and fatty acid profile of experimental diets was performed following the Association of Official Analytical Chemists procedures (25) as follows: in brief, DM after drying at 105°C until constant weight; ash by incineration in a muffle furnace at 450°C for 16 h; protein content (*N* × 6.25) by the Kjeldahl method after acid digestion using a Kjeltec digestion and distillation units (models 1015 and 1026, respectively; Tecator Systems, Sweden); gross energy by direct combustion in an adiabatic bomb calorimeter (Parr model 6200; Parr Instruments, United States); and lipid content by petroleum diethyl ether extraction (Soxtec HT System; Höganäs, Sweden). Fatty acid methyl esters were prepared by transmethylation of total lipids extract by adding sodium methylate reagent and sequentially esterification with boron trifluoride in methanol according to Bondia-Pons et al. (26) and analyzed by chromatography using Shimadzu GC-2010 Plus gas chromatograph (Shimadzu Europe GmbH, Germany) equipped with a fame-ionization detector (GC-FID) and an Omegawax 250 capillary column (30 m × 0.25 mm i.d. × 0.25 μm flm thickness; Supelco, Bellefonte, USA).

### Enzyme Activities

Liver and intestine samples (*n* = 12 per treatment) were diluted to 1:9 and 1:6, respectively, and homogenized at pH 7.8 in ice-cold 100 mM Tris–HCl buffer containing 0.1 mM EDTA and 0.1% (v/v) Triton X-100. Homogenates were centrifuged at 30,000 × g for 30 min at 4°C and the resultant supernatants were separated in aliquots and stored at −80°C for further enzyme assays. All procedures were performed on ice and all enzyme activities were measured at 37°C in a Multiskan GO microplate reader (Model 5111 9200; Thermo Scientific, Nanjing, China).

Antioxidant enzymes were analyzed as follows: superoxide dismutase (SOD; EC 1.15.1.1) activity was measured at 550 nm by the ferricytochrome C method using xanthine/xanthine oxidase as a source of superoxide radicals (27); catalase (CAT; EC 1.11.1.6) activity was determined according to Aebi (28) by measuring the decrease of hydrogen peroxide concentration at 240 nm; glutathione reductase (GR; EC 1.6.4.2) activity was determined at 340 nm by measuring the oxidation of NADPH as described by Morales et al. (29); glutathione peroxidase (GPX; EC 1.11.1.9) activity was assayed as described by Flohé and Günzler (30); the oxidized glutathione (GSSG) generated by GPX was reduced by GR and the NADPH consumption rate was monitored at 340 nm. Glucose-6-phosphate dehydrogenase (G6PDH; EC 1.1.1.49) activity was assayed by monitoring the changes in absorbance of NADP at 340 nm Morales et al. (31). Protein concentration was determined according to Bradford (32), using a commercial kit (Sigma protein Kit, cod. B6916) and bovine serum albumin as standard.

For SOD, one unit of enzyme activity was defined as the amount of enzyme necessary to produce 50% inhibition of the ferricytochrome C reduction rate. All other enzyme activities were expressed as units (CAT) or milliunits (G6PD, GPX, and GR) per milligram of hepatic soluble protein (specific activity). One unit of enzyme activity was defined as the amount of enzyme required to transform 1 μmol of substrate per minute under the assay conditions.

### Lipid Peroxidation

Malondialdehyde (MDA) concentration was used as a marker of LPO level in the liver and intestine, following the methodology described by Buege and Aust (33). An aliquot of supernatant from the homogenate (50 μL) was mixed with 250 μL of a previously prepared solution containing 15% (w/v) TCA, 0·375% (w/v) thiobarbituric acid (TBA), 80% (v/v) HCl 0.25 N, and 0.01% (w/v) butylated hydroxytoluene. The mixture was heated to 100 °C for 15 min. After being cooled to room temperature and centrifuged at 1,500 g for 10 min, the absorbance was measured at 535 nm in the supernatant. MDA concentration was expressed as nmol MDA per g of tissue, calculated from a calibration curve.

### Total and Oxidized Glutathione

Liver and intestine portions were homogenized (1:9 and 1:5, respectively), in an ice-cold solution containing 1.3% 5-sulfosalicylic acid (w/v) and 10 mM HCl, and the whole procedures were undertaken on ice to avoid glutathione oxidation. Homogenates were centrifuged at 14,000 × g for 10 min at 4°C and the resulting supernatants stored at −80°C. Total glutathione (tGSH) and oxidized glutathione (GSSG) were measured according to Pérez-Jiménez et al. (34). Standard curves of reduced glutathione (GSH) and GSSG were used for tGSH and GSSG calculations, respectively. GSH level was calculated by subtracting GSSG from tGSH values. Results are expressed as nmol/g tissue. The oxidative stress index (OSI) was calculated as: OSI = 100 × (2 × GSSG/tGSH).

### Statistical Analysis

Data are presented as mean and SEM. All data were checked for normality and homogeneity of variances and normalized when appropriate. Data were analyzed by one-way ANOVA. Significant differences among means (*p* ≤ 0.05) were determined by the Tukey's multiple range test. Statistical analyses were performed using SPSS for Windows version 25 software package.

## Results

Proximate composition was similar among diets; however, the dietary fatty acid profile was slightly different, with the microalgae diets having a higher *n*−6 polyunsaturated fatty acid (PUFA) content and an *n*−6/*n*−3 ratio than the control (Table 2).

The growth performance of the fish fed the experimental diets was not the aim of the present study, and results will be presented elsewhere. Briefly, there were no differences in growth performance between fish fed the microalgae-based diets; however, compared to the control, dietary inclusion of microalgae led to a significant decrease in growth performance (daily growth index: control 1.44 ± 0.16; Nanno15 0.92 ± 0.10; Chlo15 0.88 ± 0.14%) as well as in voluntary feed intake (control 51.9 ± 3.78; Nanno15 38.4 ± 3.81; Chlo15 37.2 ± 2.94 g/kg average body weight/day) and feed efficiency (control 0.78 ± 0.04; Nanno15 0.60 ± 0.02; Chlo15 0.60 ± 0.03).

The antioxidant enzyme activities, LPO value, glutathione content, and OSI were significantly different between the liver and the intestine. Moreover, there were significant interactions between target tissues and diets and therefore data are presented separately for each tissue.

Liver antioxidant enzyme activities are presented in Table 3. SOD, CAT and GR activities were not affected by diet composition, while GPX activity was lower in fish fed the microalgae diets than the control. G6PDH activity was also lower in fish fed the microalgae diets, though it was only statistically significantly different between the control and Chlo diets. Liver LPO, tGSH, GSH, GSSG, and OSI were unaffected by diet composition (Table 4).

**Table 3:** Specific activities of glucose-6-phosphate dehydrogenase (G6PDH), glutathione peroxidase (GPX), glutathione reductase (GR) (mU/mg protein), catalase (CAT), and superoxide dismutase (SOD) (U/mg protein) in the liver of sea bass fed the experimental diets.

	**Diets**				**One-way ANOVA**
	**Control**	**Nanno15**	**Chlo15**	**SEM**	***p-value***
CAT	285	285	309	13.1	ns
G6PDH	204^b^	163^ab^	155^a^	7.54	^*^
GR	31	31	35	1.97	ns
GPX	26^b^	17^a^	14^a^	1.20	^***^
SOD	346	345	416	14.8	ns

*Values presented as means (n = 12) and pooled standard error of the mean (SEM)*.

*Different letters in the same row stand for statistical differences between diets (p ≤ 0.05)*.

*ns, non-significant (p > 0.05)*;

* *p ≤ 0.05*;

*** *p ≤ 0.001*.

**Table 4:** Lipid peroxidation (LPO) (nmol malondialdehyde/g tissue), total glutathione (tGSH), reduced glutathione (GSH), oxidized glutathione (GSSG) (nmol/ g tissue), and oxidative stress index (OSI) in the liver of sea bass fed experimental diets.

	**Diets**				**One-way ANOVA**
	**Control**	**Nanno15**	**Chlo15**	**SEM**	***p-value***
LPO	16	18	15	0.68	ns
tGSH	2,113	1,925	2,050	56.2	ns
GSH	1,965	1,792	1,913	14.5	ns
GSSG	149	90	139	54.7	ns
OSI	14.2	9.2	14.6	1.39	ns

*Values presented as means (n = 12) and pooled standard error of the mean (SEM)*.

*ns, non-significant in the same row stands for non-statistical differences between diets (p > 0.05)*.

Intestine antioxidant enzyme activities are presented in Table 5. CAT, G6PDH, GR, and GPX activities were not affected by diet composition, but SOD activity was lower in fish fed the microalgae diets than the control. Intestine LPO, tGSH, GSH, and OSI were not affected by diet composition, but the intestine GSSG content of fish fed the microalgae diets was higher than that of fish fed the control diet(Table 6).

**Table 5:** Specific activities of glucose-6-phosphate dehydrogenase (G6PDH), glutathione peroxidase (GPX), glutathione reductase (GR) (mU/mg protein), catalase (CAT), and superoxide dismutase (SOD) (U/mg protein) in the intestine of sea bass fed the experimental diets.

	**Diets**				**One-way ANOVA**
	**Control**	**Nanno15**	**Chlo15**	**SEM**	***p-value***
CAT	209	166	173	11.4	ns
G6PDH	7.2	5.0	9.7	1.29	ns
GR	277	232	239	11.8	ns
GPX	7.2	11.6	10.0	1.34	ns
SOD	320^b^	233^a^	229^a^	14.2	^**^

*Values presented as means (n = 12) and pooled standard error of the mean (SEM)*.

*Different letters in the same row stand for statistical differences between diets (p ≤ 0.05)*.

*ns, non-significant (p > 0.05)*;

** *p ≤ 0.01*.

**Table 6:** Lipid peroxidation (LPO) (nmol malondialdehyde/g tissue), total glutathione (tGSH), reduced glutathione (GSH), oxidized glutathione (GSSG) (nmol/ g tissue), and oxidative stress index (OSI) in the intestine of sea bass fed experimental diets.

	**Diets**				**One-way ANOVA**
	**Control**	**Nanno15**	**Chlo15**	**SEM**	***p-value***
LPO	26.9	35.2	27.4	2.03	ns
tGSH	586	791	729	65.5	ns
GSH	542	724	655	5.32	ns
GSSG	44^a^	68^b^	76^b^	61.9	^**^
OSI	16	19.2	19.3	1.44	ns

*Values presented as means (n = 12) and pooled standard error of the mean (SEM)*.

*Different letters in the same row stand for statistical differences between diets (p ≤ 0.05)*.

*ns, non-significant (p > 0.05)*;

** *p ≤ 0.01*.

## Discussion

LPO was shown to be a sensitive biomarker of increased oxidative stress as it is a major result of oxidative damage induced by different types of stressors, including nutritional factors (35).

In fish, studies addressing the effect of microalgae as a protein source on tissue LPO damages are scarce. To the authors' knowledge, only the present study and the work of Qiao et al. (18) assessed such oxidative stress biomarker in fish. Accordingly, Qiao et al. (18) observed in turbot that dietary inclusion of 10% *Nannochloropsis* sp. replacing 15.5% of FM led to a reduction in plasma and liver LPO contents. The authors speculated that such protective effects of dietary microalgae against LPO in serum and liver were related to the presence of various classes of natural antioxidants (such as carotenoids, tocopherols, and phenolic compounds) in these ingredients (18, 19). Contrarily, in the present study, dietary inclusion of 15% *Nannochloropsis* or *Chlorella* did not reduce LPO content in the liver and intestine of sea bass. It is worth to notice that in the study of Qiao et al. (18) a FM-based diet was used as control while in the present study the control diet was rich in PF, and therefore the putative beneficial effects of microalgae on LPO content might have been masked due to antioxidant constituents/components present in PF.

The studies conducted so far on the effects of microalgae as a protein source on the fish antioxidant enzyme activities are inconsistent. Replacement of 10–20% FM with the microalgal biomass of *Desmodesmus sp*. in Atlantic salmon did not alter serum CAT and SOD activities (19). Rahimnejad et al. (21) found similar effects for plasmatic GPX and SOD activities, but not for CAT activity, that increased when olive flounder juveniles were fed diets containing 10–15% of defatted *Chlorella vulgaris*. Sorensen et al. (20) reported an increase in serum SOD activity of Atlantic salmon fed a diet with 10% *N. oceania*-derived defatted meal compared to that in fish fed on algae-devoid diet, but such increase was not observed in fish fed 20% alga-containing diet. Qiao et al. (18) also found that activities of the antioxidant enzymes GPX and SOD in the liver and plasma of turbot increased with dietary *Nannochloropsis* sp. inclusion up to 5%, but decreased at higher dietary levels. In the present study, GPX activity was also decreased in the liver of fish fed the microalgae-rich diets. As GPX is the main enzyme involved in quenching lipid-peroxidizing chain reactions, this may indicate decreased LPO in the liver. The reduction of lipid peroxides to their corresponding alcohols or hydrogen peroxide to water by GPX typically uses glutathione (GSH) as a reducing agent, leading to the production of its oxidized form (GSSG) which is then regenerated to GSH by GR. In this process, NADPH is oxidized. NADPH is produced in the pentose phosphate pathway, being G6PDH the rate-limiting enzyme in the process (7). Despite the action of GPX, GR, and G6PDH are mechanistically associated, only G6PDH activity in the Chlo group followed as expected the decrease in GPX activity.

To the authors' knowledge, the impact of dietary microalgae on the intestinal antioxidant mechanisms in fish is limited to a study in gilthead sea bream (*Sparus aurata*) fed practical diets supplemented with 1.5% of *Nannochloropsis gaditana* (36), where no signs of nutritional regulation of CuZn- and Mn-SOD and CAT were observed at the transcriptional level. Even so, there is crescent evidence that microalgae may be of value to counteract or prevent the impairment of intestinal health generally observed in fish fed aquafeeds rich in PF. Indeed, Grams et al. (37) observed that dietary FM replacement by 20% *Chlorella vulgaris* was effective to counteract the intestinal inflammation observed in Atlantic salmon fed a diet containing 20% soybean meal. In the present study, although no improvement on LPO levels in the intestine was noticed, dietary microalgae inclusion may have contributed to decreasing ROS production, as it is suggested by the decrease of SOD activity. Nonetheless, although depletion of tGSH and GSH pools were not observed in fish fed the microalgae diets, the changes in GSSG content at intestinal level suggest a higher risk of oxidative stress.

Overall, the results of this study suggest that activation of antioxidant enzymatic mechanisms was not necessary for fish fed the microalgae-rich diets and that, at least at the intestinal level, non-enzymatic antioxidant mechanisms (namely glutathione) sufficed to prevent increased oxidative damage. This may probably represent a less energy-demanding process. Thus, the lack of induction of the antioxidant enzyme mechanisms may be regarded as potentially beneficial for fish fed microalgae diets.

Our results also showed that the antioxidant defense strategies were tissue-dependent, possibly due to the different susceptibility to oxidative stress of each organ. The intestine showed an overall higher peroxidation potential (higher LPO content) than the liver, possibly due to its high cell turnover and continuous exposition to potentially toxic or allergenic compounds from the diets (such as ANF) that are prone to ROS (38). At the intestinal level, the antioxidant defense strategy to deal with the high generation of oxyradicals involved a tight regulation of the non-enzymatic antioxidant defense mechanism (glutathione) and increased SOD activity, the enzyme that acts as the first line of the enzymatic defense system against ROS.

On the other hand, while the intestine is a major consumer of GSH, the liver is thought to be the main organ for GSH synthesis and export (39, 40). Moreover, as the liver is among the main lipid storage organ in sea bass, it may be more at risk from lipid peroxidative attack than the intestine. Therefore, signaling pathways that are more sensitive to lipid peroxides, as GPX-related pathways are more prone to be activated at hepatic than at intestinal levels.

Overall, the results of the present study indicate that dietary replacement of FM by *Chlorella* sp. or *Nannochloropsis* sp. modulate the liver and intestine antioxidant enzyme response without detrimental effects on overall lipid oxidative damage. These results are encouraging for understanding the potential role of microalgae in modulating fish redox state in the context of using microalgae as alternative protein sources in sea bass diets. But, future work should consider a deep characterization of bioactive compounds in microalgae biomass in order to identify the biological activity of these compounds and ascertain the mechanisms involved in the microalgae-modulator role in fish antioxidant mechanisms.

## Data Availability Statement

The raw data supporting the conclusions of this article will be made available by the authors, without undue reservation.

## Ethics Statement

The animal study was reviewed and approved by ORBEA (Orgão de Bem-Estar e Ética Animal/Animal Welfare and Ethics Body) of CIIMAR and the Portuguese National Authority for the Animal Health (DGAV).

## Author Contributions

CC and FC carried out the main experimental work and wrote the draft of the manuscript under the direction of the project designer and leaders AC and AO-T. PI supplied microalgae biomass. All authors contributed to the article and approved the manuscript.

## Conflict of Interest

PI was employed by the company Buggypower Lda. The remaining authors declare that the research was conducted in the absence of any commercial or financial relationships that could be construed as a potential conflict of interest.
